# Ultrasound Imaging in Football Players with Previous Multiple Ankle Sprains: Keeping a Close Eye on Superior Ankle Retinaculum

**DOI:** 10.3390/bioengineering11050419

**Published:** 2024-04-25

**Authors:** Carmelo Pirri, Nina Pirri, Diego Guidolin, Veronica Macchi, Andrea Porzionato, Raffaele De Caro, Carla Stecco

**Affiliations:** 1 Department of Neurosciences, Institute of Human Anatomy, University of Padova, 35121 Padova, Italy; diego.guidolin@unipd.it (D.G.); veronica.macchi@unipd.it (V.M.); andrea.porzionato@unipd.it (A.P.); rdecaro@unipd.it (R.D.C.); carla.stecco@unipd.it (C.S.); 2 Department of Medicine—DIMED, School of Radiology, Radiology Institute, University of Padova, 35121 Padova, Italy; nina_92_@hotmail.it

**Keywords:** ankle, retinacula, deep fascia, muscular fascia, soccer, sport, ankle sprain

## Abstract

The superior extensor ankle retinaculum (SEAR), a transversely fascial thickening positioned above the tibia–talar joint, serves as a crucial anatomical structure in ankle stability. The purpose of this study was to measure and compare by ultrasound (US) imaging the bilateral thickness and echogenicity of SEAR in football players with previous multiple ankle sprains (group 1) and healthy volunteers (group 2). A cross-sectional study was performed using ultrasound imaging to measure longitudinal and transversal axes using a new protocol in a sample of 50 subjects: 25 football players with previous multiple ankle sprains and 25 healthy subjects. The findings for SEAR thickness revealed statistically significant differences for both axes (*p* = 0.0011 and *p* = 0.0032) between the healthy side and the previously sprained side, and with the corresponding side of group 2 (*p* = 0.003 and *p* = 0.004). Moreover, in group 1, regarding the ROI echogenicity, a statistically significant difference was found between the sides (*p* = 0.0378). These findings suggest that the football players with previous ankle sprains showed a thicker and inhomogeneous SEAR on the sprain side, unveiling a remodeling of this structure compared to the other side and to the healthy volunteers. In these athletes, during US examination, one needs to keep “a US eye” on side-to-side SEAR comparisons.

## 1. Introduction

Football, with its global appeal, attracts a vast audience of around 270 million participants, ranging from enthusiastic to seasoned professionals [1]. Among them, 110,000 make football their profession, while 38 million engage in organized leagues [2,3]. An additional 226 million play for recreational purposes, enjoying the physical and health benefits [2,3]. Success in football hinges on a combination of individual skills, technical proficiency, tactical understanding, and teamwork [2,3]. Football players in successful teams showcase superior physical abilities, including specific physiological and neuromuscular skills. Football, a fast-paced and energetic sport, involves various movements such as sprinting, changing direction, ball skills, jumping, and physical contact, all of which contribute to a high risk of injury [2,3]. Various studies reported injury rates in professional football ranging from 0.5 to 45 injuries per 1000 h of exposure to match and training environments [4,5,6,7].

Most injuries affect the knee, ankle, thigh, and calf muscles and ligaments, with lower extremity injuries being particularly prevalent among male professional players, occurring at rates between 61% and 90% [2,3,8,9,10]. Approximately 66% of football injuries are classified as traumatic, while the remaining third are attributed to overuse [2,3,9]. In high-level football, ankle injuries represent a significant portion, ranging from 10% to 18% of all reported injuries [11,12,13]. Among these, ankle sprains are the most prevalent, constituting between 51% and 81% of all ankle injuries [13,14,15]. These sprains predominantly affect the lateral ligaments and are typically sustained during player-to-player contact situations, constituting 59% of cases [16,17]. Remarkably, for goalkeepers, a notable 79% of ankle sprains occur in non-contact scenarios [16]. Additionally, Jain et al. [17] reported a recurrence rate of 28.6% for the anterior talofibular ligament. 

Some studies reported that injuries of the retinacula are associated with ankle sprains [18,19,20], but the dearth of extensive studies on the topic renders a comprehensive analysis challenging. Typically, these injuries stem from forceful and abrupt stretching of the neighboring tendons triggered by significant muscular contractions [18]. The ankle retinacula can be effectively assessed via MRI, as underscored by Numkarunarunrote et al. [19] and Stecco et al. [20]. These structures manifest as bands of low signal intensity, typically measuring around 1 mm in thickness, yet a comprehensive characterization of potential alterations remains elusive [19,20]. Ultrasound (US) imaging of the ankle retinacula is particularly effective because of their superficial position and its high spatial resolution [21,22,23]. Moreover, dynamic ultrasound examination can optimally show an intermittent tendon dislocation related to retinacular failure [24]. The superior extensor ankle retinaculum (SEAR), which is a transversely rectangular band and a fascial reinforcement positioned above the tibia–talar joint, serves as a crucial anatomical structure. It attaches laterally to the anterior border of the distal tibia and the lateral malleolus, and medially to the anterior tibial crest and the medial malleolus [20]. It has demonstrated considerable variation in both width and thickness [20] and a crucial role in the ankle stability [20]. 

Therefore, we aimed to determine an ultrasonographic parameter or difference that can quantify the superior extensor ankle retinaculum status in football players with previous multiple ankle sprains compared with healthy volunteers. 

## 2. Materials and Methods

### 2.1. Study Design

A cross-sectional study, adhering to the Strengthening the Reporting of Observational Studies of Epidemiological (STROBE) guidelines, was undertaken [25] to assess the ultrasound thickness and echogenicity of SEAR in male football players with a history of previous multiple ankle sprains, in comparison with healthy volunteers. The research adhered to the principles outlined in the Helsinki Declaration and complied with ethical guidelines of human experimentation [26]. Approval for the study was obtained from the Ethics Committee of the University of Padova. Prior to participation, all participants were provided with written consent forms approving the research and informing them about the study.

### 2.2. Participants

A total of 50 subjects were recruited and categorized into two groups: “group 1” consisted of 25 football players with a history of previous multiple ankle sprains while “group 2” comprised 25 healthy volunteers, from March 2020 to February 2024. The inclusion criteria for participation in group 1 required football players to have experienced at least two episodes of grade II ankle sprains within the past 5 years, with no occurrences in the previous 6 months, along with a confirmation of ankle ligament integrity through physical examination and ultrasound imaging. In group 2, the inclusion criteria mandated no history of ankle sprains or any limiting ankle pain or instability during daily activities. The exclusion criteria for both groups encompassed a history of lower limbs or ankle surgery, ankle or foot deformities, severe ankle or foot pain, previous fractures of ankle or foot bones or lower extremities, balance disorders, and systemic disease such as rheumatological conditions, diabetes, etc. Healthy volunteers in group 2 were matched with football players in terms of age, sex, and BMI. All participants underwent ultrasound examination to evaluate the ultrasound thickness of the superior extensor ankle retinaculum. 

### 2.3. Ultrasound Examination Measurements

Employing an advanced high-resolution ultrasound machine (Edge II, Sonosite, FUJIFILM, Inc. 21919, Washington, WA, USA) equipped with a frequency range of 6–15 MHz and featuring a screen resolution of 1680 × 1050 pixels, ultrasound images were acquired at precise anatomical landmarks surrounding the ankle. These landmarks medially included the anterior crest of the tibia and the medial malleolus, and laterally the lateral crest of the distal fibula and the surface of the lateral malleolus, adhering to a specific ultrasound protocol. The ultrasound assessment was conducted by a physician specialist in physical and rehabilitation medicine with 8 years of expertise in skeletal muscle and fasciae ultrasound examinations. A standardized protocol was developed and adhered to for the bilateral assessment of the SEAR. The ultrasound system operated at a speed of sound of c = 1540 m/s, a standard setting for diagnostic ultrasound machines, and was configured to B-mode with a depth of penetration set at 30 mm. Adequate amounts of gel were applied by the ultrasonographer to facilitate optimal scanning and minimize surface pressure on the skin. Care was taken to place the probe gently on the skin to avoid tissue compression while ensuring stable contact to produce consistent images. 

To mitigate the impact of potential variations in tissue thickness, three equidistant points per image of the SEAR were measured, and the resulting values were averaged for analysis. The ultrasonographer adhered to a consistent protocol to ensure the uniform quantification of each point of the SEAR. The ultrasound beam was maintained perpendicular to the SEAR to mitigate anisotropy artifacts that commonly affect it. The power and overall gain of the US machine were meticulously maintained at the same settings for all evaluations. The ultrasound scanner configurations remained consistent throughout the duration of the study. Subsequently, the ultrasound images were captured and frozen for further analysis. 

**Figure 1:**
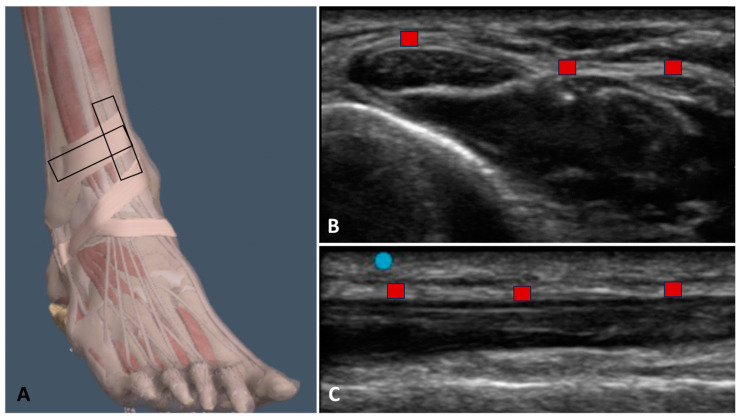
Ultrasound measurement protocol of SEAR thickness. (**A**) Starting from the transversal axis to the longitudinal axis, rotating by 90° degrees and sliding medially to laterally to acquire three points of evaluation on the longitudinal axis. (**B**) Transversal axis. (**C**) Longitudinal axis.

Firstly, the ultrasonographer used the short axis, as it offers optimal visualization to facilitate tracking of the landmarks associated with the ultrasound imaging of the SEAR, as suggested by Pirri et al. [24]. Subsequently, the ultrasonographer rotated the probe by 90° degrees to perform the longitudinal axis assessment. A specific protocol was established at the mid-third of the leg near the ankle. The subject was positioned supine, with relaxation ensured, and the ultrasound transducer was positioned parallel to the tibia, approximately 0.5 cm lateral to the medial tibial crest (see Figure 1), above the tibia–talar joint, and lateral to the anterior border of the distal tibia up to the lateral malleolus. Scans were conducted along the short axis, meticulous attention being paid to maintaining a consistent structure at the center of the ultrasound monitoring image, and ensuring the probe remained perpendicular. Subsequenlty, rotation of the probe by 90° degrees was performed for the long axis assessment; moreover, after taking the longitudinal scan at this point, the probe was slid medially and laterally so as to evaluate three regions of the SEAR. 

At the conclusion of each ultrasound assessment, all images were halted and saved, and the thickness and echogenicity of the SEAR was quantified using the Image J analysis software (accessible online: https://imagej.nih.gov/ij/, accessed on 5 February 2024). Each image was partitioned into three sections, within which three points offering optimal visibility were gauged and then averaged. To mitigate the potential impact of thickness discrepancies, three equidistant points within each image were measured, and the resultant values were averaged for analytical purposes. The echogenicity exhibited by the SEAR was evaluated in the transversal scans. For this purpose, the entire SEAR was interactively defined by grey levels, in which the value for each pixel could range from 0 (black) to 255 (white) [27]. In real units, every pixel corresponded to 0.1 mm. Consequently, after careful US evaluation by the ultrasonographer, identifying points of US alteration of the SEAR structure, a region of interest (ROI) of 4.5 mm^2^ within the SEAR structure of the previously sprained side and a corresponding one on the healthy side were interactively defined and subjected to grey level analysis, in which the value for each pixel could range from 0 (black) to 255 (white) [27]. The mean grey level exhibited by the entire SEAR and the particular ROI were considered as an estimator of its echogenicity. 

### 2.4. Statistical Analysis

Statistical analysis was conducted utilizing GraphPad PRISM 8.4.2 (GraphPad Software Inc., San Diego, CA, USA), with a significance threshold set at *p* < 0.05. Effect size was determined using G Power 3.1 (Universtität Düsseldorf: Psychologie) and interpreted according to Cohen’s kappa as small (d = 0.20), medium (d = 0.50), and large (d = 0.80) [28]. In our pilot study and as supported by another study [20], the effect size for the SEAR thickness was calculated as d = 1, with an α error probability of 0.05, a 1-β error probability of 0.95 (power), and a sample size of 23 for each group [20]. However, our study included a sample of 25 individuals for every group. A normality assessment was conducted using the Kolmogorov–Smirnov test. Descriptive statistics, including measures of central tendency and dispersion ranges, were calculated for both groups separately using the mean and standard deviation (SD) to describe parametric data. Subsequently, a comparative analysis between the football player and the healthy volunteer groups was conducted by employing an unpaired Student’s *t*-test. Differences in US-estimated SEAR thickness across various axes were statistically analyzed using a paired Student’s *t*-test. Differences in estimated echogenicity between the different sides and groups were subjected to statistical analysis using one-way analysis of variance (ANOVA) followed by Tukey’s test for multiple comparisons. Furthermore, Pearson’s test was utilized for both groups to evaluate the correlation between BMI, weight, height, age, and SEAR thickness. Additionally, a two-way, mixed-model, intra-class correlation coefficient (ICC 3, k) type C was employed to assess intra-rater reliability. The interpretation of ICC values categorized them as poor when below 0.5, moderate when between 0.5 and 0.75, good when between 0.75 and 0.9, and excellent when above 0.90 [29]. 

## 3. Results

A total of 50 subjects (50 males) participated in this study. The descriptive data of the sample are summarized in Table 1. No differences were present for BMI, height, weight, or age, showing homogeneity among the groups.

### 3.1. Ultrasound Measurements of the Superior Extensor Ankle Retinaculum

#### 3.1.1. Group 1 (Football Players with Previous Multiple Ankle Sprains)

Regarding Table 2, the SEAR in the football players with previous multiple ankle sprains had a mean ultrasound thickness of 1.3 ± 0.5 mm on the previous ankle sprain side and 0.9 ± 0.4 mm on the healthy side. 

Moreover, for both sides, according to the paired Student’s *t*-test, the comparison between longitudinal and transversal axis thicknesses showed no statistically significant difference: healthy side (long.) vs. healthy side (transv.), *p* = 0.76; previously sprained side (long.) vs. previously sprained side (transv.), *p* = 0.81. 

#### 3.1.2. Group 2 (Healthy Volunteers)

In the healthy volunteers, the SEAR thickness was 0.9 ± 0.45 mm (Table 3).

Moreover, for both sides, according to the paired Student’s t-test, the comparison between the longitudinal and transversal axis thicknesses showed no statistically significant difference: right side (long.) vs. right side (transv.), *p* = 0.54; S. side (long.) vs. S. side (transv.), *p* = 0.96. 

### 3.2. Ultrasound Measurements of the Superior Extensor Ankle Retinaculum: Comparison between Previous Multiple Ankle Sprains and Healthy Sides in Group 1

**Figure 2:**
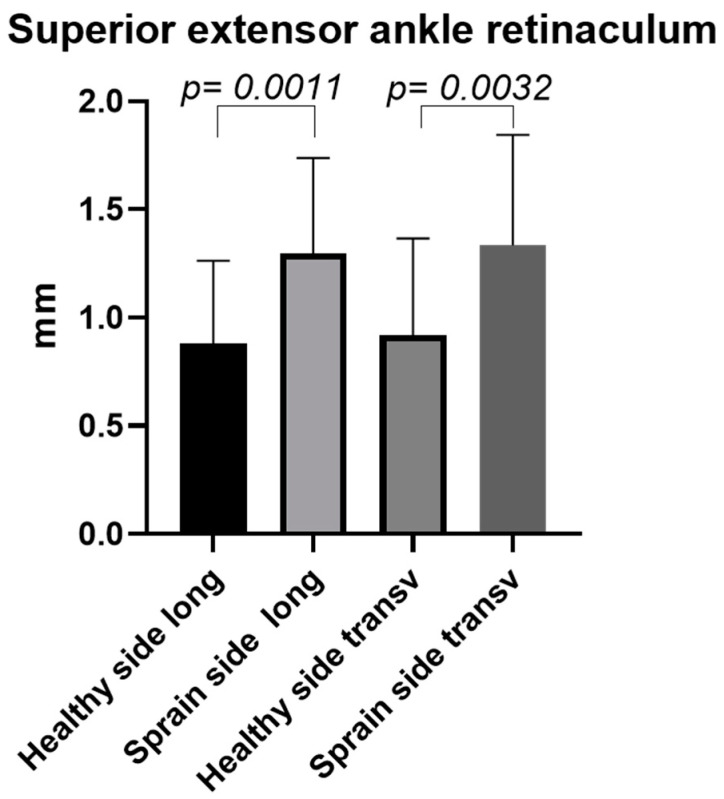
Group 1: Ultrasound thickness measurements of SEAR in football players with previous multiple ankle sprains. Long., longitudinal; transv., transversal.

**Figure 3:**
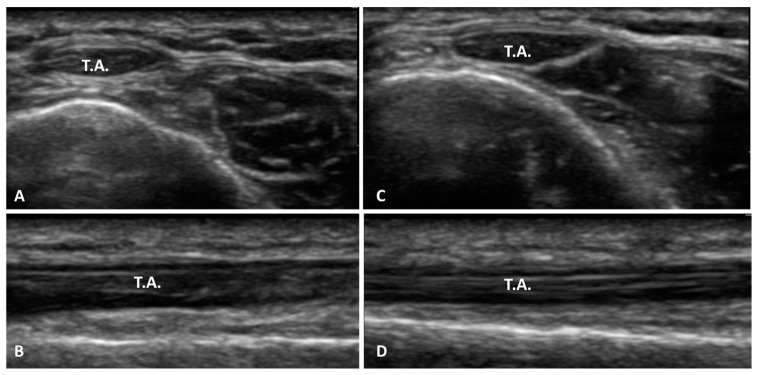
Ultrasound images of SEAR thickness: (**A**) healthy side group 1: transversal axis; (**B**) healthy side group 1: longitudinal axis; (**C**) sprain side group 1: transversal axis; (**D**) sprain side group 1: longitudinal axis. T.A., tibialis anterior tendon.

The comparison between the different sides (previous ankle sprain side vs. healthy side) showed a statistically significant difference in the SEAR ultrasound thickness (Figure 2 and Figure 3). These differences were present for both the longitudinal axis and the transversal axis; respectively, *p* = 0.0011 and *p* = 0.0032. 

### 3.3. Ultrasound Measurements of the Superior Extensor Ankle Retinaculum: Comparison between Two Sides in Group 2

**Figure 4:**
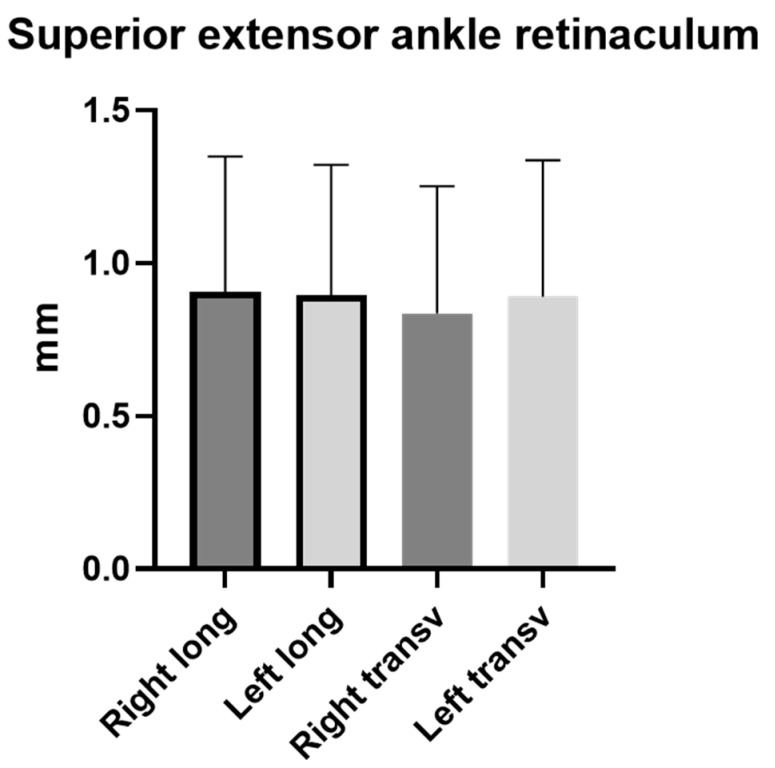
Group 2: Ultrasound thickness measurements of SEAR in healthy volunteers. Long.: longitudinal; transv.: transversal.

According to the paired Student’s *t*-test, the comparison between the right side and the left side showed no statistically significant difference (Figure 4); group 2 left long. vs. group 2 right long.: *p* = 0.92; group 2 left transv. vs. group 2 right transv.: *p* = 0.53. 

### 3.4. Ultrasound Measurements of the Superior Extensor Ankle Retinaculum: Comparison between the Previously Sprained Side of Group 1 with the Corresponding Side in Group 2

According to the unpaired Student’s *t*-test, the comparison between the previously sprained side of group 1 with the corresponding side in group 2 showed a statistically significant difference in the SEAR US thickness. These differences were present for both the longitudinal and transversal axes (Table 4).

### 3.5. Ultrasound Measurements of the Superior Extensor Ankle Retinaculum: Comparison between the Healthy Side of Group 1 with the Corresponding Side in Group 2

The comparison between the healthy side in group 1 and the corresponding side in group 2 showed no statistically significant difference in the ultrasound SEAR thickness for both the longitudinal and transversal axes (Table 5). 

### 3.6. Echogenicity Measurements

Regarding Table 6, the entire SEAR echogenicity in the football players had a mean of 94.61 ± 27.17 on the sprain side and 93.84 ± 29.10 on the healthy side, whereas in the healthy volunteers, the echogenicity had a mean of 80.80 ± 29.04 on the right side and 89.44 ± 26.72 on the left side (Table 6). 

According to Tukey’s multiple comparisons test (Table 7), the comparison between the echogenicity on the different sides and between the two groups showed no statistically significant difference.

**Figure 5:**
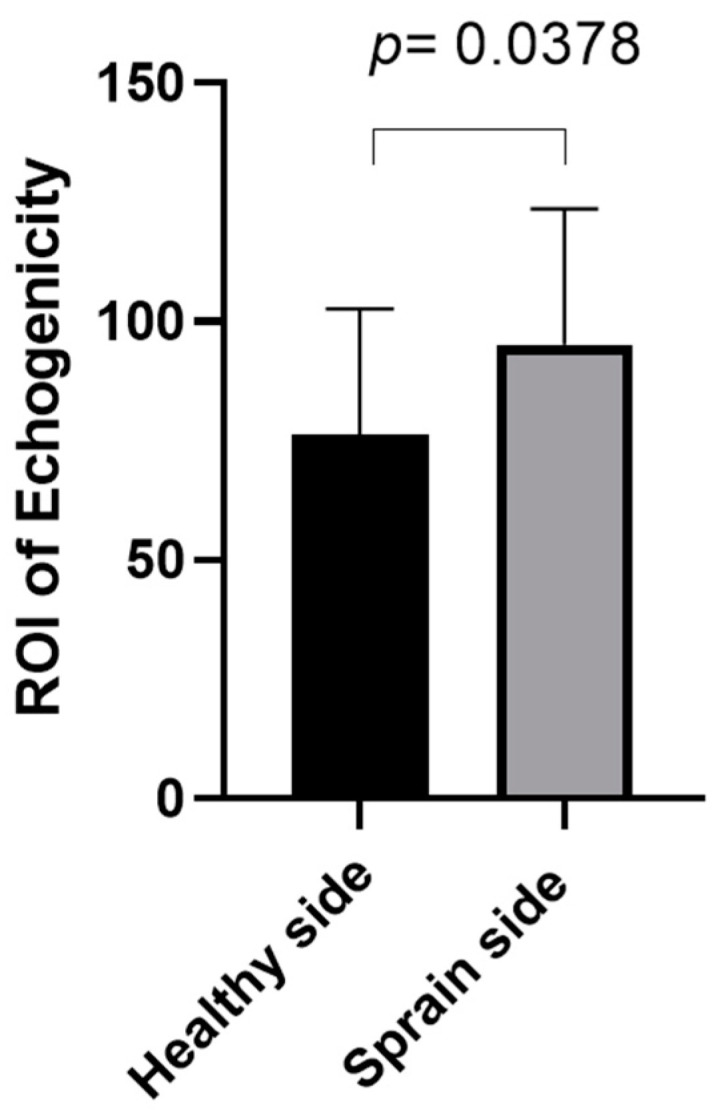
Group 1: ROI echogenicity measurements of SEAR in healthy side and sprain side.

In group 1, the comparison between the ROI identified by the ultrasonographer as an area of SEAR structure alteration and the corresponding one on the healthy side showed a statistically significant difference (Figure 5).

### 3.7. Correlations US Measurements and Number of Ankle Sprains

**Figure 6:**
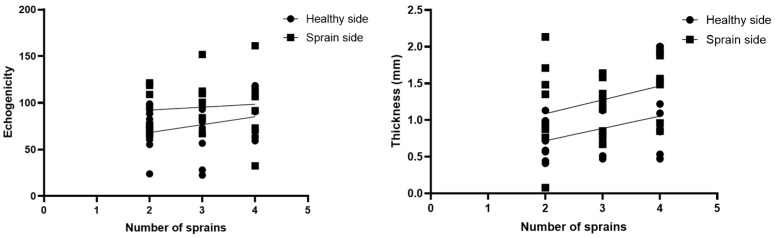
Group 1: correlations between number of ankle sprains and echogenicity and thickness of SEAR in sprain side.

According to the correlation analysis, there was no statistically significant correlation between the numbers of ankle sprains and the SEAR thickness of the sprain side (r = 0.3213; *p* = 0.1173) or the SEAR echogenicity (r = 0.09273; *p* = 0.6593) (Figure 6).

### 3.8. Intra-Rater Reliability

In addition, the intra-rater reliability was reported as good and excellent. The results for the longitudinal SEAR were as follows: healthy side longitudinal axis for group 1: ICC_3,k_: 0.90 (0.89–0.94); healthy side transversal axis for group 2: ICC_3,k_: 0.92 (0.88–0.96); previous ankle sprain side longitudinal axis for group 1: ICC_3,k_: 0.91 (0.88–0.94); previous ankle sprain side transversal axis for group 1: ICC_3,k_: 0.92 (0.88–0.96); right side longitudinal axis for group 2: ICC_3,k_: 0.90 (0.89–0.94); right side transversal axis for group 2: ICC_3,k_: 0.91 (0.89–0.94); left side longitudinal axis for group 2: ICC_3,k_: 0.90 (0.89–0.94); left side transversal axis for group 2: ICC_3,k_: 0.91 (0.89–0.96) (Table 8). 

## 4. Discussion

Based on our current knowledge, this study may be described as the first study detailing the SEAR thickness and echogenicity in football players with previous multiple ankle sprains compared with healthy volunteers with the same characteristics in terms of height, weight, and age. As has been reported by other studies assessing the SEAR, the SEAR was visualized in the transversal and longitudinal axes, appearing with multilayer, linear, and hyperechogenic layers below the subcutaneous tissue [18,21]. The primary aim of this study was to examine the difference in the thickness of the SEAR between football players with previous multiple ankle sprains and healthy volunteers. An analysis of our results of the SEAR thickness showed that in group 1, along the longitudinal and transversal axes, it was thicker on the previous multiple ankle sprains side (long. = 1.3 ± 0.44 mm; transv. = 1.33 ± 0.51 mm) than on the healthy side (long. = 0.9 ± 0.4 mm; transv. = 0.92 ± 0.44 mm) (Table 2), showing statistical differences for both the longitudinal axis and the transversal axis—respectively, *p* = 0.0011 and *p* = 0.0032 (Figure 2 and Figure 6)—whereas in the healthy volunteers, the SEAR thickness along the longitudinal and transversal axes of the right and left sides was right: long. = 0.90 ± 0.44 mm; transv. = 0.83 ± 0.42 mm; left: long. = 0.90 ± 0.43 mm; transv. = 0.90 ± 0.44, showing no statistically significant differences (Table 3 and Figure 3).

In light of these findings, the SEAR thickness tended to be thicker on the previous multiple ankle sprains side, underlying the importance of the side-to-side ultrasound examination of ankle retinacula. This aspect bears significant clinical relevance, as it would enable the identification of any alterations in force transmission within the fascial system. Over time, the SEAR underwent structural modifications in reaction to recurring stresses induced by pre-existing aberrant movement patterns resulting from repetitive motions, habitual postures, and athletic activities [20]. Moreover, the increased SEAR thickness was related to the transmission of myofascial forces; it can undergo significant alterations in terms of stiffness and impairment of movement, transforming it into compromised tissue characterized by densification and fibrosis [20]. These findings corroborate previous studies [20,22,23], indicating that cases of chronic injuries, microinjuries, and/or inflammation impact body movement patterns through a complex network of interconnected mechanisms, including abnormal sensory input and maladaptive tissue remodeling [30]. Additionally, statistically significant differences in the SEAR thickness were evident between football players and healthy volunteers when comparing the corresponding side to the side with previous/recurrent ankle sprains of football players along the longitudinal and transversal axes; respectively, *p* = 0.003 and *p* = 0.004. 

Moreover, the echogenicity evaluations of the entire SEAR showed no statistically significant differences, neither between the sprain side and healthy side in group 1 nor between group 1 and group 2. However, the comparison between the echogenicity evaluation of the ROI identified by the ultrasonographer as an expression of SEAR structure alteration and the corresponding one on the healthy side showed a statistically significant difference. 

Ankle retinacula represent localized thickening of the deep fascia, which are structured during development, due to movement [31]. They serve to secure tendons in position during muscle contraction, permitting gliding between them [20]. Given the considerable mechanical strain experienced by the ankle during athletic activities, such as in football, along with multiple acute traumas and repetitive microtraumas, their increase in thickness and ROI echogenicity could be common in clinical practice, albeit likely underreported in the literature. According to our ROI echogenicity data, the football players with previous/recurrent ankle sprains had a remodeling of their SEAR, but this remodeling was not homogenous and was evident at particular points. Pirri et al. [30] demonstrated that the mechanobiology of fasciae respond to particular molecular pathways that are related to precise biophysical stimuli, and myofascial pathways of force are closely related to various directions of movement used during specific sports tasks [2,3,30]. An alteration of the viscoelastic features of the deep fascia of the foot and lower limbs and their joint and ligamentous structures, which are in anatomical continuity with them, can modify the field lines of traction, tension, and compression within the SEAR, creating particular points of alteration in their structure. Moreover, the anatomical continuity between the ankle retinacula and the deep fascia of the lower limbs elucidates why other authors have consistently observed changes in motor neuron pool excitability in muscles that operate on joints proximal to the ankle in individuals with chronic multiple ankle sprains [20]. That being said, the alteration of the muscle activation patterns in recurrent ankle sprains modifies the proprioception of these football players in a cascade [2,3,21]. 

Ultrasound imaging proved particularly adept at visualizing the SEAR due to its superficial position and the technology’s high spatial resolution [18,21,22,23]. Pirri et al. [22] reported that expertise in ultrasound imaging and identifying anatomical landmarks from a fascial point of view is crucial, as is the position of the probe and the type of axis; for this reason, we decided to create this protocol for the SEAR. 

Furthermore, the lack of correlation between the number of ankle sprains and the SEAR thickness and echogenicity could be explained by the fact that after previous multiple ankle sprains, the SEAR becomes densified or fibrotic in a particular pattern of movements; “a vicious cycle” was established, which after an initial increase in thickness and echogenicity becomes an integral part of the football players’ posture and sports tasks, feeding on itself and preferring particular direction patterns, without the deposition of a new extracellular matrix. 

These findings have corroborated previous reports from other studies regarding excellent intra-rater reliability when assessing the deep muscular fasciae using ultrasound imaging [32]. This reliability is particularly notable when the sonographers possess proficient technical skills in ultrasound and a deep understanding of fascial anatomy [32]. 

To the best of our knowledge, this is the inaugural study to assess and compare SEAR thickness and echogenicity across various axes using ultrasound imaging in both football players with previous multiple ankle sprains and healthy volunteers. Future longitudinal investigations with larger football player cohorts hold promise for enriching our understanding of the pathophysiology underlying diverse thickness and echogenicity patterns. Moreover, ultrasound imaging may unveil alterations that are imperceptible during routine clinical examinations. Ultimately, delineating SEAR thickness and echogenicity in cases of retinacula dysfunctions could “pave” the way for more precise and tailored treatment and prevention strategies 

### Limitation of Study

Despite having good power, this study should be expanded to a large population of football players to investigate the frequency of observed ultrasound findings and elucidate their potential origins, prognostic importance, and therapeutic implications. Additionally, the assessment of SEAR morphology via ultrasound heavily relies on the expertise of the sonographer and the appropriate configuration of the US device.

## 5. Conclusions

Ultrasound imaging offers an optimal means for visually assessing ankle retinacula, in particular the SEAR, in football players with previous multiple ankle sprains. It stands out as a safe, cost-effective, non-invasive, portable, and notably efficacious tool that can enhance clinicians’ understanding of retinacula dysfunction and pathology. Additionally, it has the potential to reveal subtle changes overlooked by standard clinical examinations, some of which warrant further investigations as they have yet to be thoroughly documented. In summary, the findings of this study affirmed a statistically significant alteration in thickness and echogenicity in the ankles of football players with previous multiple ankle sprains, pointing out altered retinacula remodeling that maintained an inhomogeneous and thicker SEAR compared to the other ankle and to healthy volunteers. The differences found underline the importance of establishing standardized landmarks to perform side-to-side assessments of the SEAR and to make results comparable. 

## Figures and Tables

**Table 1:** Descriptive data of the sample.

Data	Group 1	Group 2	*p*-Value Group 1 vs. Group 2
Age, year	29.96 ± 10.54	28.09 ± 12.38	*p* = 0.14
Weight, kg	69.22 ± 6.1	72.60 ± 12.20	*p* = 0.45
Height, cm	174.3 ± 4.83	171.30 ± 6.76	*p* = 0.55

Mean ± standard deviation (SD) was applied.

**Table 2:** Ultrasound thickness (mm) measurements of the SEAR in football players with previous multiple ankle sprains. Abbreviations: long., longitudinal scan; transv., transversal scan; H., healthy; S., previous multiple sprains.

Descriptive Statistics	H. Side (long.)	H. Side (trans.)	S. Side (long.)	S. Side (trans.)
Number of values	25	25	25	25
Minimum	0.41	0.44	0.67	0.50
Maximum	2	1.72	2.14	2.51
Mean	0.90	0.92	1.3	1.33
Std. deviation	0.4	0.44	0.44	0.51
Std. error of mean	0.08	0.08	0.08	0.1

**Table 3:** Ultrasound thickness (mm) measurements of the SEAR in healthy volunteers. Abbreviations: long., longitudinal scan; transv., transversal scan; R., right; L., left.

Descriptive Statistics	R. Side (long.)	R. Side (transv.)	L. Side (long.)	L. Side (transv.)
Number of values	25	25	25	25
Minimum	0.41	0.43	0.43	0.44
Maximum	2.1	2.1	1.9	1.9
Mean	0.90	0.83	0.90	0.90
Std. deviation	0.44	0.42	0.43	0.44
Std. error of mean	0.08	0.08	0.08	0.08

**Table 4:** Ultrasound SEAR thickness (mm) measurement comparison between group 1 and group 2. Abbreviations: long., longitudinal scan; transv., transversal scan; H., healthy; S., previous multiple ankle sprains; c.s., corresponding side.

Type of Comparison	Mean Diff.	*p*-Value
Group 1 S. side (long.) vs. group 2 c.s. (long.) Group 1 S. side (transv.) vs. group 2 c.s. (transv.)	0.3902 0.4981	*p* = 0.003 *p* = 0.004

**Table 5:** Ultrasound SEAR thickness (mm) measurements comparison between the healthy side of group 1 and the corresponding side in group 2. Abbreviations: long: longitudinal scan; transv: transversal scan; H.: healthy; S.: previous multiple ankle sprains; c.s.: corresponding side.

Type of Comparison	Mean Diff.	*p*-Value
Group 1 H. side (long.) vs. group 2 c.s. (long.) Group 1 H. side (transv.) vs. group 2 c.s. (transv.)	−0.045 0.027	*p* = 0.68 *p* = 0.82

**Table 6:** Echogenicity measurements of the SEAR in football players (group 1) and healthy volunteers (group 2). Abbreviations: H., healthy; S., sprain; R., right; L., left.

Descriptive Statistics	H. Side (Group 1)	S. Side (Group 1)	R. Side (Group 2)	L. Side (Group 2)
Number of values	25	25	25	25
Minimum	36.58	45.05	46.2	41.91
Maximum	166.1	158.9	145.7	133.8
Mean	93.84	94.61	80.80	89.44
Std. deviation	29.10	27.17	29.04	26.72

**Table 7:** Comparison in echogenicity measurements of the SEAR between sides in the groups and between football players (group 1) and healthy volunteers (group 2). Abbreviations: H., healthy; S., sprain; c.s., corresponding side.

Type of Comparison	Mean Diff.	*p*-Value
Group 1 H. side vs. group 1 S. side	−0.77	*p* = 0.99
Group 1 H. side vs. group 2 c.s.	13.04	*p* = 0.35
Group 1 H. side vs. group 2 c.s.	4.39	*p* = 0.94
Group 1 S. side vs. group 2 c.s.	13.81	*p* = 0.31
Group 1 S. side vs. group 2 c.s.	5.16	*p* = 0.91
Group 2 right vs. group 2 left	−8.64	*p* = 0.69

**Table 8:** Intra-rater reliability of the ultrasound SEAR thickness measurements within different axes of group 1 and group 2. Abbreviations: H., healthy; S., sprain; R., right; L., left.

Type of Axis	ICC
Group 1 H. side (long.)	0.90 (0.89–0.94)
Group 1 H. side (transv.)	0.92 (0.88–0.96)
Group 1 S. side (long.)	0.91 (0.88–0.94)
Group 1 S. side (transv.)	0.92 (0.88–0.96)
Group 2 left (long.)	0.90 (0.89–0.94)
Group 2 left (transv.)	0.91 (0.89–0.96)
Group 2 right (long.)	0.90 (0.89–0.94)
Group 2 right (transv.)	0.91 (0.89–0.94)

## Data Availability

The data presented in this study are available upon request from the corresponding authors. The data are not publicly available due to privacy.
